# Effects of resveratrol on bone health in type 2 diabetic patients. A double-blind randomized-controlled trial

**DOI:** 10.1038/s41387-018-0059-4

**Published:** 2018-09-20

**Authors:** Simona Bo, Roberto Gambino, Valentina Ponzo, Iolanda Cioffi, Ilaria Goitre, Andrea Evangelista, Giovannino Ciccone, Maurizio Cassader, Massimo Procopio

**Affiliations:** 1 Department of Medical Sciences, University of Turin, Turin, Italy; 2 Department of Clinical Medicine and Surgery, Federico II University Hospital, Naples, Italy; 3 Unit of Clinical Epidemiology, CPO, “Città della Salute e della Scienza” Hospital of Turin, Turin, Italy

## Abstract

**Objectives:**

Patients with type 2 diabetes (T2DM) are at increased fracture risk. Resveratrol has shown beneficial effects on bone health in few studies. The aim of this trial was to investigate the effects of resveratrol on bone mineral density (BMD) and on calcium metabolism biomarkers in T2DM patients.

**Methods:**

In this double-blind randomized placebo-controlled trial 192 T2DM outpatients were randomized to receive resveratrol 500 mg/day (Resv500 arm), resveratrol 40 mg/day (Resv40 arm) or placebo for 6 months. BMD, bone mineral content (BMC), serum calcium, phosphorus, alkaline phosphatase, and 25-hydroxy vitamin D were measured at baseline and after 6 months.

**Results:**

At follow-up, calcium concentrations increased in all patients, while within-group variations in alkaline phosphatase were higher in both resveratrol arms, and 25-hydroxy vitamin D increased in the Resv500 arm only, without between-group differences. Whole-body BMD significantly decreased in the placebo group, while whole-body BMC decreased in both the placebo and Resv40 arms. No significant changes in BMD and BMC values occurred in the Resv500 arm. The adjusted mean differences of change from baseline were significantly different in the Resv500 arm vs placebo for whole-body BMD (0.01 vs −0.03 g/cm^2^, *p* = 0.001), whole-body BMC (4.04 vs −58.8 g, *p* < 0.001), whole-body T-score (0.15 vs −0.26), and serum phosphorus (0.07 vs −0.01 µmol/L, *p* = 0.002). In subgroup analyses, in Resv500 treated-patients BMD values increased to higher levels in those with lower calcium and 25-hydroxy vitamin D values, and in alcohol drinkers.

**Conclusions:**

Supplementation with 500 mg resveratrol prevented bone density loss in patients with T2DM, in particular, in those with unfavorable conditions at baseline.

## Introduction

Type 2 diabetes (T2DM) subjects are at a higher risk for bone fracture due to altered bone cell function and bone remodeling, advanced glycation end-product (AGE) accumulation causing collagen deterioration, and microarchitectural changes^1,2^.

The list of the healthy properties of the polyphenolic compound resveratrol (3,5,4ʹ-trihydroxy-trans-stilbene), although not all confirmed in human studies^3–6^, has recently lengthened after the discovery of the benefits on bone metabolism for this compound^7^. Animal studies have shown that resveratrol prevents bone loss, reduces mineral density due to immobilization, older age, and ovariectomy^8–11^, and causes bone healing and repair after surgical procedures or trauma^12,13^. It promoted osteoblastogenesis, antagonized osteoclasts in vitro^14–16^, and potentiated vitamin D receptor activity^17^. However, other preclinical studies reported mixed or negative effects of resveratrol on bone health^18–21^.

Despite this substantial preclinical evidence, human data about the effects of resveratrol on bone metabolism are very scarce^22,23^. An increase in circulating levels of bone-specific alkaline phosphatase after 4 weeks of resveratrol supplementation (compared to placebo) has been described in a small trial in obese men^22^. Resveratrol has shown to increase lumbar spine trabecular volumetric bone mineral density (BMD) in a dose-dependent manner in middle-aged, obese men with metabolic syndrome^23^.

We have recently found no metabolic or anti-inflammatory benefits of resveratrol^24^, but an increment in the concentrations of antioxidant markers and pentraxin-3^25^ in patients with T2DM. In this study therefore, we have investigated the effects of resveratrol at dosages of 40 and 500 mg/day for 6 months on BMD and on the circulating concentrations of calcium metabolism biomarkers in these patients by a double-blind randomized placebo-controlled trial.

## Subjects and methods

### Participants

Participants were enrolled at the Diabetic Clinic of the Department of Medical Sciences of the University of Turin, between October 2013 and February 2016. Procedures have been previously reported^24,25^.

Inclusion criteria were: diagnosed T2DM patients, age ≥ 40 years, body mass index (BMI) < 35 kg/m^2^, patients on diet and/or hypoglycemic agents other than insulin. Exclusion criteria were: treatment with any antioxidant substance, treatment with insulin, anticoagulants, steroids, or anti-inflammatory drugs different from aspirin, alcohol or substance abuse, uncompensated diabetes, liver or kidney diseases, presence of diabetes-related chronic complications, cardiovascular events or revascularization procedures in the previous four weeks, or any severe chronic or life-threatening diseases, pregnancy, allergy to peanuts, grapes, wine, mulberries.

### Study design

The present study was a double-blind, randomized, placebo-controlled trial, and has been registered in December 2011 at clinicaltrials.gov (ID NCT01492114).

### Intervention

As previously described^24^, 192 patients were randomized to 1 capsule/day of resveratrol 500 mg/day (Resv500 arm) for 6 months, 1 capsule/day of resveratrol 40 mg/day (Resv40 arm) for 6 months and 1 capsule/day of placebo (totally inert microcellulose) for 6 months (placebo arm) (Supplemental Fig. 1). All participants were asked to assume one capsule/day in the morning and to maintain their habitual lifestyle, the diet given by the Diabetic Clinic, and their current hypoglycemic treatment during the trial. The use of antioxidant nutritional supplements or consuming significant amounts of resveratrol-rich foods and beverages were not allowed during the trial.

### Compliance

Compliance with the study protocol was monitored with monthly phone calls and pill counting.

All the laboratory measurements were blind and performed at the Laboratory of Metabolic Diseases of the Department of Medical Sciences, University of Turin.

### Outcomes

The primary outcome of the present study was to measure the difference in changes in BMD values from baseline to end of trial in patients treated with resveratrol 500 mg/day or resveratrol 40 mg/day compared to patients treated with placebo.

Secondary outcomes were the differences in changes from baseline to trial end of BMC, serum values of calcium, phosphorus, alkaline phosphatase (ALP), 25-hydroxy vitamin D in patients treated with resveratrol 500 mg/day or resveratrol 40 mg/day compared to patients treated with placebo.

### Randomization

A computer-generated randomization sequence was developed by a statistician using blocks of various length in random sequence, and patients were stratified by use of acetyl-salicylic acid and glycated hemoglobin (HbA1c) levels (cut-point 7%)^24^. The procedure was concealed to researchers and available on a dedicated web site (www.epiclin.it).

### Blinding

The bottles containing resveratrol and placebo capsules were identical and were prepared by a person who did not take part in the study and were labeled with patient identification number. Patients and researchers dispensing the capsules and involved in the trial were blinded to the bottle content. All laboratory and densitometric analyses were blind procedures.

### Ethical aspects

All procedures were in agreement with the principles of the Helsinki Declaration; the study protocol was approved by the Local Ethics Committee. All participants provided written informed consent to participate in the study.

### Measurements

At baseline and after 6 months (trial end), data related to health status, the use of drugs or supplements, and usual dietary habits and exercise levels were collected from all subjects.

Patients had to complete a validated food-frequency questionnaire^24^ and the Minnesota-Leisure-Time-Physical-Activity questionnaire^26^.

All the following measurements were collected at baseline and after 6 months: body weight, waist circumference, arterial blood pressure, Dual X-ray densitometry (DXA) measurements of BMD, bone mineral content (BMC), and fat percentage. Furthermore, fasting blood samples were collected to determine the circulating concentrations of metabolic, and inflammatory variables, serum calcium, phosphorus, ALP, and 25-hydroxy vitamin D.

Alcohol intake was assessed by multiplying the mean daily intake for each beverage by its ethanol content, to give grams of alcohol/day. Level of physical activity was calculated as the product of the duration and frequency of each activity (in hours/week), weighted by an estimate of the metabolic equivalent (MET) of activity and summed for activities performed^24^.

The measurements and the laboratory assays have been described elsewhere^24^.

BMD was measured by DXA (QDR-4500; Hologic, Bedford, MA, USA); areal BMD and BMC at the lumbar spine (L1-L4), hip, and whole body were measured by DXA using the same instrument at baseline and at the trial end. Coefficient of variation of repositioning was <1.5% for lumbar spine and total hip, and <2% for whole body.

DXA was used to determine lean and fat body mass, using whole-body absorptiometry software.

The lumbar, hip, and whole-body BMD T-scores were calculated according to the following formula: (patient’s BMD − mean young-adult BMD) / (SD young-adult BMD).

Blood samples, collected after an overnight fast, were centrally analyzed.

A direct colorimetric method for determination of serum calcium and phosphorus was used (Sentinel, Milan) with intra-assay and inter-assay variation coefficients (CVs) of 0.4–0.9 and 1.5–1.7% (calcium) and 0.7–1.2 and 2.2–4.4% (phosphorus), respectively. An enzymatic colorimetric method was used for determination of serum ALP (Sentinel, Milan) according to the DGKC recommendations with an intra-assay and inter-assay CVs of 0.8–2.1 and 1.6–1.9%. 25-hydroxy vitamin D was determined by competitive solid phase enzyme linked immunosorbent assay (Global Diagnostic B, Belgium). The kit has a sensitivity of 2.6 ng/ml in a 25-µL sample size and a range of 0 to 105 ng/ml. The intra-assay and inter-assay CVs were, respectively, 3.6–8.6 and 6.4–7.7%.

### Statistical methods

The sample size was originally calculated for another outcome, the reduction of C-reactive protein (CRP): to obtain an effect size of 0.50^27^, a total sample size of 192 patients (about 64 per arm) was necessary to reach a statistical power of 80% considering an overall type 1 error of 5%^24^.

Within-group comparisons were analyzed by *t*-test for paired samples or Wilcoxon-matched paired test, as appropriate.

Analysis of covariance (ANCOVA) was used to compare changes from baseline of the analyzed endpoints between the resveratrol and placebo arms, adjusted for the baseline endpoint measurement, stratification variables used in the randomization (aspirin use and HbA1c concentrations), gender, and use of calcium/vitamin D supplements. For each endpoint, a global test was performed to evaluate if at least one of two resveratrol arms showed significantly different changes with respect to the placebo arm.

To preserve the overall 5% type 1 error, a gatekeeping strategy was adopted accounting for the hierarchical structure of multiple comparisons in the first step. The Resv500 arm was first compared with placebo and only if this test was statistically significant at *p* < 0.05, a comparison between Resv40 arm and placebo with a test *p* < 0.05 was considered as significant.

Exploratory subgroup analyses were performed to identify potential interactions with the experimental treatment according to patient characteristics (age, gender, exercise levels expressed by METs, baseline percent body fat mass, baseline calcium, or 25-hydroxy vitamin D levels), disease conditions (diabetes duration, HbA1c values), and other variables (aspirin or statin use, smoking, alcohol consumption, and hypoglycemic drugs). Subgroup analyses were adjusted for the all same variables used for adjusting the ANCOVA analyses (aspirin use, HbA1c, gender, use of calcium/vitamin D supplements).

Statistical analyses were performed using Stata 13.2 software (StataCorp LP, College Station, Texas).

## Results

Median age of the enrolled patients was 66 years (interquartile range 60–70); 66% were males; all females were in menopause. Mean BMI and percent body fat were, respectively, 28.8 ± 3.9 kg/m^2^ and 32.3 ± 7.6%; these values as well as dietary intakes and exercise levels were similar among the three arms^24^.

Out of the 192 patients, 65 were randomized to the Resv500 arm, 65 to the Resv40 arm, and 62 to the Placebo arm. At the trial end, 3, 6, and 4 patients, respectively, dropped out and data of 62, 59, and 58 patients from Resv500, Resv40, and Placebo arms were available (Supplemental Fig. 1).

More than 95% compliance was detected through pill counting; no serious adverse event occurred.

Calcium/vitamin D supplements were taken by 2/3/3 patients, respectively, in the Resv500, Resv40 and Placebo arms, whereas the corresponding proportions of individuals treated with metformin, sulfonylureas, and incretin drugs were respectively 67.7, 35.4, 26.2% (Resv500), 67.7, 32.3, 29.2% (Resv40), and 66.1, 37.1, 24.2% (Placebo). No participant was treated with thiazolidinediones or gliflozins.

Baseline variables were homogenous for placebo and the resveratrol arms (Table 1), however, the proportion of female participants in the Resv40 group was higher and was a casual occurrence, as no gender stratification had been planned.

**Table 1:** Baseline variables by arm of the trial

	Placebo arm	Resv40 arm	Resv500 arm	P*
Number	62	65	65	
Age (years)	65.4 ± 8.8	64.9 ± 8.6	65.0 ± 7.6	0.94
Males (%)	75.8	58.5	63.1	0.10**
Diabetes duration (years)	8.0 (10.0)	7.0 (13.0)	7.0 (10.0)	0.98***
Glycated hemoglobin (%)	6.9 ± 1.0	7.2 ± 1.3	6.9 ± 1.2	0.33
Calcium (mmol/L)	2.07 ± 0.25	2.03 ± 0.24	2.00 ± 0.18	0.30
Phosphorus (mmol/L)	1.23 ± 0.22	1.27 ± 0.24	1.33 ± 0.26	0.06
Alkaline phosphatase (U/L)	146.4 ± 38.1	157.6 ± 45.5	142.5 ± 38.6	0.09
25 hydroxy vitamin D (ng/mL)	20.1 ± 10.5	21.6 ± 11.1	19.6 ± 8.0	0.49
BMD (g/cm^2^)				
Lumbar spine	1.03 ± 0.19	0.98 ± 0.16	0.96 ± 0.18	0.12
Total hip	0.97 ± 0.15	0.97 ± 0.12	0.95 ± 0.16	0.88
Whole body	1.08 ± 0.14	1.06 ± 0.12	1.06 ± 0.13	0.66
BMC (g)				
Lumbar spine	57.7 ± 15.2	53.0 ± 18.6	52.8 ± 16.5	0.18
Total hip	41.0 ± 10.7	38.9 ± 8.8	38.7 ± 11.0	0.39
Whole body	2326.5 ± 509.0	2205.5 ± 461.5	2204.4 ± 520.0	0.29
T-score				
Lumbar spine	−0.6 (2.1)	−1.0 (1.6)	−1.0 (2.1)	0.31***
Total hip	−0.4 (1.6)	−0.3 (1.0)	−0.4 (1.3)	0.65***
Whole body	−1.1 (1.3)	−1.5 (1.5)	−1.2 (1.8)	0.60***

*p-values obtained by ANOVA; **p-values obtained by chi-square test; ***p-values obtained by Kruskal–Wallis test

Overall, the resveratrol arms showed significantly different changes in serum phosphorus, whole-body BMD and whole-body BMC with respect to the placebo arm. In detail, the adjusted mean differences of change from baseline significantly differed only in the Resv500 arm versus Placebo: phosphorus (0.07 vs −0.01 µmol/L, respectively, Resv500 and Placebo groups), whole-body BMD (0.01 vs −0.03 g/cm^2^), whole-body BMC (4.04 vs −58.8 g), whole-body T-score (0.15 vs −0.26) (Table 2).

**Table 2:** Comparisons on change from baseline of the analyzed variables

	Placebo arm		Resv40 arm				Resv500 arm				P*
	Trial end	Within-group mean change	Trial end	Within-group mean change	Adjusted mean difference on change β 95% CI	*P*	Trial end	Within-group mean change	Adjusted mean difference on change β 95% CI	*P*	
Number	58		59				62				
Males (%)	77.6		62.9				64.4				
Calcium (mmol/L)	2.17 ± 0.36	0.11	2.15 ± 0.36	0.12	0.00 −0.15, 0.14	0.95	2.18 ± 0.34	0.18	0.02 −0.14, 0.18	0.84	0.79
Phosphorus (mmol/L)	1.21 ± 0.19	−0.01	1.28 ± 0.27	0.02	0.04 −0.04, 0.12	0.32	1.40 ± 0.28	0.07	0.13 0.05, 0.21	0.002	0.007
Alkaline phosphatase (U/L)	152.1 ± 47.6	7.1	168.8 ± 56.1	10.0	2.80 −9.88, 15.5	0.66	153.2 ± 41.6	10.7	4.37 −6.94, 15.7	0.45	0.76
25 hydroxy vitamin D (ng/mL)	20.9 ± 10.5	0.58	22.1 ± 10.8	0.39	0.53 −3.17, 4.23	0.78	23.8 ± 8.7	4.00	3.45 0.29, 6.61	0.03	0.14
BMD (g/cm^2^)											
Lumbar spine	1.04 ± 0.24	0.005	0.98 ± 0.16	0.004	0.00 −0.03, 0.03	0.96	0.97 ± 0.19	0.01	0.01 −0.02, 0.05	0.42	0.68
Total hip	0.96 ± 0.17	−0.006	0.98 ± 0.14	0.01	0.02 −0.00, 0.04	0.06	0.96 ± 0.18	0.001	0.01 −0.01, 0.03	0.30	0.25
Whole body	1.06 ± 0.13	−0.03	1.07 ± 0.14	−0.01	0.02 −0.00, 0.04	0.11	1.07 ± 0.13	0.01	0.03 0.02, 0.05	<0.001	0.006
BMC (g)											
Lumbar spine	57.4 ± 16.1	−0.33	53.9 ± 19.9	0.37	0.81 −1.47, 3.08	0.48	52.2 ± 16.9	−0.22	−0.12 −2.68, 2.44	0.93	0.78
Total hip	41.5 ± 11.0	−0.07	39.8 ± 11.5	0.11	0.14 −1.05, 1.33	0.81	38.9 ± 11.5	0.15	0.34 −0.62, 1.29	0.49	0.90
Whole body	2292.3 ± 501.1	−58.8	2203.7 ± 454.3	−42.9	18.16 −16.9, 53.3	0.31	2210.5 ± 526.5	4.04	63.8 30.9, 96.7	<0.001	<0.001
T-score											
Lumbar spine	−0.6 (2.4)	−0.07	−1.0 (1.8)	0.007	0.07 −0.06, 0.21	0.28	−0.9 (2.2)	0.01	0.08 −0.03, 0.19	0.16	0.43
Total hip	−0.5 (1.9)	−0.03	−0.2 (1.1)	−0.04	0.00 −0.07, 0.06	0.94	−0.4 (1.2)	0.005	0.04 −0.05, 0.13	0.38	0.51
Whole body	−1.4 (1.5)	−0.26	−1.5 (1.7)	−0.18	0.13 −0.04, 0.29	0.14	−1.2 (1.5)	0.15	0.19 0.04, 0.34	0.01	0.07

Analyses were performed by using ANCOVA (adjusted for the baseline level, gender, the stratification variables, and the use of calcium/vitamin D supplements). Trial end values were reported as mean ± SD or median (interquartile range. Within-group mean change were obtained as: (trial end value – baseline value)). Adjusted mean difference on change were obtained from the comparisons between the difference between the change in the resveratrol arms (either Resv40 or Resv500 arm) and the change in the placebo arm (ANCOVA coefficients)

*p-values for the global test among the three arms, obtained by ANCOVA to evaluate if at least one of two resveratrol arms showed significantly different changes with respect to the placebo arm

In all the three arms, calcium concentrations increased after 6-month follow-up, but mean changes were not significantly different between groups. Within-group changes in ALP were higher in both resveratrol arms, and 25-hydroxy vitamin D and whole-body T-score in the Resv500 group, only. Whole-body BMD and whole-body T-score significantly decreased within the Placebo group, while whole-body BMC values were reduced within both the Placebo and the Resv40 arms. No significant changes in BMD and BMC values were observed in the Resv500 arm.

Data did not meaningfully change after excluding the 8 patients on calcium/vitamin D supplements, and after adjusting for duration of diabetes and HbA1c values.

**Fig. 1:**
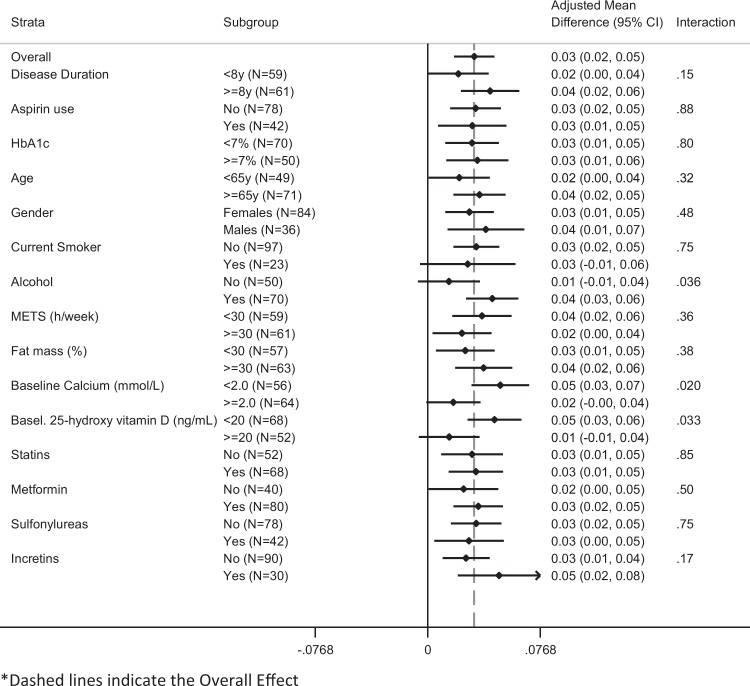
Adjusted mean difference on change from baseline (95% CI) of total BMD values (Resv500 arm vs Placebo arm)

**Fig. 2:**
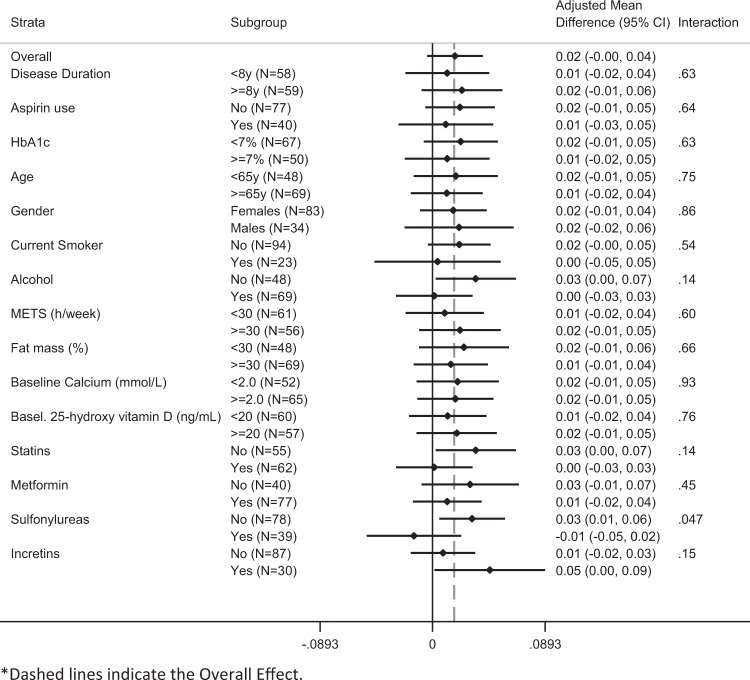
Adjusted mean difference on change from baseline (95% CI) of total BMD values (Resv40 arm vs Placebo arm)

Subgroup analyses were performed to analyze potential factors which could have modified the effects of resveratrol administration (Figs. 1 and 2). There was no heterogeneity of effects for the evaluated variables in the adjusted mean difference on changes from baseline of BMD values in the Resv40 arm vs the Placebo arm, except in the case of patients treated with sulfonylureas (Fig. 2). BMD values increased in almost all subgroups of the Resv500 arm when compared to the Placebo arm (Fig. 1). A statistically significant interaction for BMD was noted relatively to alcohol intake, baseline calcium and 25-hydroxy vitamin D concentrations, since BMD values increased more in patients with alcohol intake, and in those with lower than median values of calcium and 25-hydroxy vitamin D (Fig. 1).

## Discussion

Resveratrol supplementation was associated with positive effects on bone density in patients with T2DM, and particularly in those with unfavorable conditions at baseline.

Animal and in vitro studies have shown a protective effect of resveratrol on bone status by the prevention of bone mass reduction and downregulation of bone turnover thus preserving bone structure, volume, microarchitecture, and mechanical strength; it also enhances bone formation and growth, by antagonizing osteoclasts, promoting osteogenic differentiation and osteoblast activity, and decreasing the bone marrow mesenchymal stem cell differentiation into adipocytes, mediated by the activation of Sirtuin-1 (SIRT-1)^8–13,15,28,29^.

The presumed mechanism by which resveratrol may exert its beneficial effects on the bone are: increased bone sulfhydryl content and reduction in bone pro-inflammatory mediators (malondialdehyde, IL-6, TNFα, etc), VEGF, and oxidative stress (which is involved in the development of osteoporosis), the hypothesized estrogenic activity, suppression of the upregulation of mRNA levels of Peroxisome proliferator-activated receptor-γ (PPARγ), restoration of bone mRNA levels of insulin-like growth factor 1 (IGF-1) and Wnt/β-catenin signaling pathway, upregulation of the expression of osteo-lineage genes RUNX2, and upregulation of the gene expression of bone morphogenetic proteins^8–13,30,31^. Furthermore, the suppression of the release of bone pro-inflammatory cytokines by resveratrol may reduce receptor activator for NF-κB ligand (RANKL) expression by osteoblasts, thus inhibiting osteoclast proliferation and bone resorption^11^.

Not all studies were consistent, as neutral or negative bone effects of resveratrol have also been described^18–21,32^. Possible explanations of these divergences may be because of the different duration of supplementation, different models, species, or experimental conditions, and the type of supplement employed.

Furthermore, the effectiveness of resveratrol is quite different when tested in in vitro or animal studies when compared to clinical trials in humans^4^.

Chronic sub-clinical inflammation and oxidative stress associated with diabetes might contribute to the unhealthy bone status of T2DM patients, together with other mechanisms, such as the increased skeletal content of advance glycation end products (AGEs), the altered differentiation of osteogenic cells, the altered bone turnover and microarchitecture^1,2^. Bone loss due to inflammation is associated with greater resorption activity by osteoclasts^8^. In our patients, the maintenance of BMD and the increase in ALP suggested that resveratrol might attenuate diminished bone formation found in T2DM patients on placebo. However, we have recently failed to detect any anti-inflammatory effects of resveratrol in the patients under study^24^. Indeed, recent animal studies showed that resveratrol has a beneficial role on osteoblasts independently of inflammation, thus being a more general bone protective agent^14^. The two currently available human studies were in line with our results. The first short-term (4 weeks) trial, performed in 24 obese non-diabetic men, 12 randomized to a high-dose (1500 mg/day) resveratrol supplementation, and 12 to placebo, found a 15% increase in bone-specific ALP and a trend in total ALP increment, not reaching statistical significance, while inflammatory markers did not change^22^. In the other clinical trial, men with metabolic syndrome were randomized for 16 weeks to 150 mg/day resveratrol (*n* = 21), 1000 mg/day resveratrol (*n* = 21), or placebo (*n* = 24), and a dose-dependent increase in bone ALP and in lumbar spine volumetric BMD were found, with no changes in inflammatory markers^23^.

In the latter study, lumbar spine bone mass and density increased in a dose-dependent manner in the resveratrol-treated men, while no consistent change at total hip and whole body was detected^23^. We have shown that the supplementation with 500 mg resveratrol prevents the reduction of BMD and BMC observed in patients treated with placebo. However, the inclusion in our sample of older T2DM individuals and menopausal women might justify these differences.

Three further considerations should be made: firstly, in a 6-month period, our non-supplemented T2DM patients showed a ~3% bone mass loss. Therefore, even if BMD values are reported to be increased in T2DM patients when compared to controls^1,2^, also in the diabetic patients within our age range a significant bone loss was evident in a relatively short period of time. Secondly, although the effect of 500 mg resveratrol on bone density was low, a ~1% increase over a 6-month intervention period, seems to be relevant, especially considering bone loss incurring in controls, and therefore worth to be tested in long-term trials. Finally, we found benefits on whole-body BMD, BMC, and T-scores, but not on lumbar spine or total hip values, which are the clinically useful measures to predict the risk of osteoporotic fractures. Indeed, in overweight/obese individuals, as are our participants and most T2DM patients, the greatest risk seems to be that of appendicular fractures^33,34^. Therefore, the beneficial effects on whole-body measures may be clinically relevant in these subjects.

Although ALP is a non-specific isoenzyme, it is considered as an acceptable marker for bone formation^11,14,22,23,28^. ALP acts as a transmembrane receptor involved in osteoprogenitor–osteoblast adhesion, migration, and differentiation, whereas mineralization is caused by coprecipitation of calcium and inorganic phosphates (generated by ALP catalyzed glycerophosphate degradation) onto a collagen matrix^35^. We have found a significant within-group increase in ALP values in the resveratrol arms, thought the differences were not significantly different from placebo.

Resveratrol shares some similar mechanisms with vitamin D, one of the regulators of calcium and phosphorus homeostasis, and these two substances show important mutual processes and interactions^36^. In particular, vitamin D, besides modulating a variety of pathways such as cell growth and division, regulation of immune responses and antimicrobial defense, xenobiotic detoxification, carcinogenesis, neuroprotection, and insulin regulation, has shown, similarly to resveratrol, to inhibit adipogenesis and stimulate osteoblastogenesis, partly because of its ability to inhibit PPARγ expression^29^. Resveratrol potentiates 1,25-dihydroxyvitamin D3-binding to the Vitamin D receptor (VDR), activates its co-receptor, the Retinoid X Receptor (RXR), and stimulates SIRT-1, an enzyme able to potentiate vitamin D signaling via VDR deacetylation^37,38^.

Accordingly, we found a slightly significant increase in 25-hydroxy vitamin D circulating levels in patients treated with 500 mg resveratrol. Moreover, calcium and phosphorus concentrations increased after resveratrol treatment, even if only the changes in phosphorus levels were significantly different in the Resv500 arm. Similarly, higher femoral phosphorous, but not calcium, was found in hindlimb-suspended rats given resveratrol^8^, and plasma calcium levels were unchanged after resveratrol supplementation in humans^22,23^. A different regulation of calcium and phosphorus absorption by vitamin D has been called into question: vitamin D enhances the absorption of phosphorus by increasing the activity of ALP brush border, which hydrolyzes phosphorus ester bonds^8^. Indeed, the beneficial effect of resveratrol on bone was reported to be vitamin D-independent^13^, and the expression pattern for osteoblast markers was different for these two substances^29^. Therefore, a specific effect of resveratrol on phosphorus metabolism could not be excluded.

The dose-effect of resveratrol was a confirmation of the causal-effect relationship between resveratrol supplementation and bone health, since the beneficial effects on all outcomes were enhanced with increasing doses of the supplement. If confirmed by further studies, the potential anabolic osteogenic properties of resveratrol might be particularly interesting for older T2DM patients, characterized by gradual bone loss due to reducing osteoblast-mediated bone formation^7^.

### Subgroup analyses

Resveratrol seemed to be more effective on BMD in subgroups with unfavorable baseline characteristics, i.e., those with lower baseline values of calcium/25 hydroxy vitamin D, and alcohol drinkers. This is intriguing, since all these conditions give to a higher risk of bone demineralization, microstructural deterioration, and fractures. Alcohol increases the risk for loss of BMD and impairment of bone remodeling, by an inhibitory effect on osteogenic differentiation of bone marrow-derived mesenchymal stem cells (BM-MSCs), inducing a premature senescence-associated phenotype in these cells, also at moderate doses^39^. We have excluded heavy alcohol drinkers from the present trial, but <30 g/day alcohol consumers have been enrolled. We found an increased benefit of resveratrol 500 mg on BMD values in alcohol consumers. Indeed, resveratrol supplementation via SIRT-1 activation was shown to be able to suppress senescence phenotypes in BM-MSCs, partially counteracting the effects of ethanol and rescuing the inhibited osteogenesis^39^.

Finally, patients in the Resv40 arm and not on sulfonylureas showed an increase in BMD. A neutral effect of sulfonylureas on the risk of fractures in T2DM patients was reported by a few observational studies, suffering from many limitations^40^. However, sulfonylureas can cause hypoglycemia, leading to an increased risk of falls and therefore to fractures, as recently demonstrated in a large cohort study^41^. Data about the incidence of hypoglycemia in our patients were not available, and thus we were not able to test this hypothesis.

### Limitations

This trial has a few limitations, such as the unfeasibility of DXA for evaluation of changes in bone geometry and microstructure; the precision error of DXA measurements increases with higher BMI; biochemical markers of bone turnover and serum parathormone levels were not measured; the determination of circulating variables does not necessarily reflect actions at the tissue level; even if our study had the longest time duration among published human trials, it could not determine the impact of resveratrol on the fracture risk, the major bone complication of T2DM patients. Furthermore, the sample size was originally calculated on CRP value reduction^24^; indeed, we estimated “a posteriori” that the present study achieved a power = 80% to detect a difference of 0.019 in BMD change from baseline between the Resv500 and the Placebo arms, with a two-tailed α-value = 0.05. Finally, the subgroup analyses were based on small groups and did not allow to obtain definitive conclusions.

However, our results were consistent, and in accordance with the existing literature. Moreover, this is the first trial evaluating the bone effects of resveratrol in T2DM patients. Other strengths included its randomized placebo-controlled double-blind design; the high patients’ adherence, and the centralized measurements.

## Conclusion

In conclusion, the supplementation with resveratrol was associated with slight beneficial effects on bone density in patients with T2DM, especially in specific high-risk subgroups of patients. Additional studies are required to demonstrate whether the continuous administration of resveratrol could reverse the increased risk of bone fractures of these patients.

## Electronic supplementary material

Flow of the study
