# Supplementary material for: Effects of resveratrol on bone health in type 2 diabetic patients. A double-blind randomized-controlled trial
Source: Nutr Diabetes. 2018 Sep 20;8:51. doi: 10.1038/s41387-018-0059-4 (PMC6147949; doi:10.1038/s41387-018-0059-4)
Supplement: Supplementary file 1 — Flow of the study [file 41387_2018_59_MOESM1_ESM.ppt]

## Slide 1
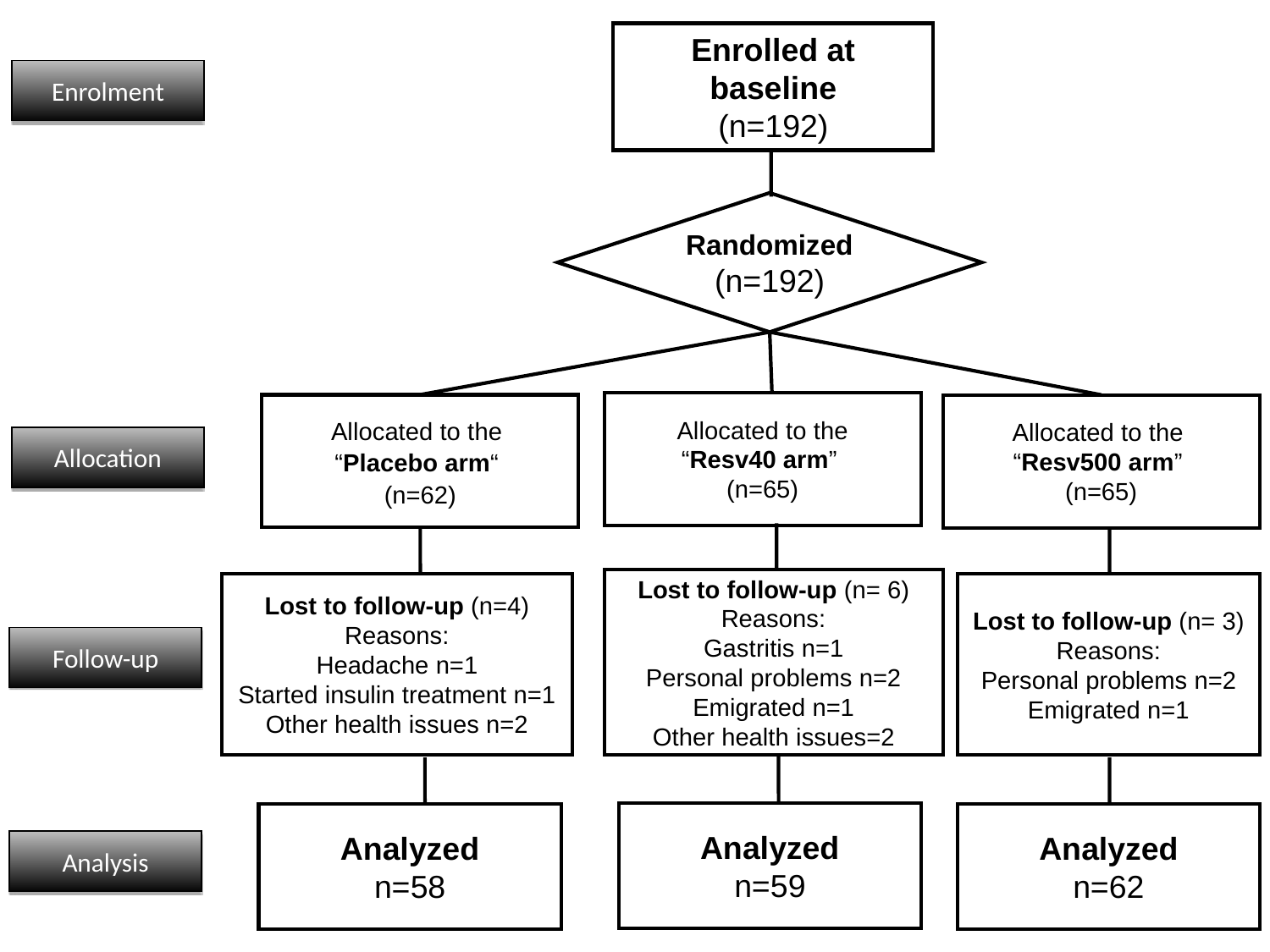

Enrolled at baseline
(n=192)
Enrolment
Randomized
(n=192)
Allocated to the
“Resv40 arm”
(n=65)
Allocated to the
“Placebo arm“
(n=62)
Allocated to the
“Resv500 arm”
(n=65)
Allocation
Lost to follow-up (n= 6)
Reasons:
Gastritis n=1
Personal problems n=2
Emigrated n=1
Other health issues=2
Lost to follow-up (n=4)
Reasons:
Headache n=1
Started insulin treatment n=1
Other health issues n=2
Lost to follow-up (n= 3)
Reasons:
Personal problems n=2
Emigrated n=1
Follow-up
Analyzed
n=59
Analyzed
n=58
Analyzed
n=62
Analysis
